# Causal effect of gut microbiota on DNA methylation phenotypic age acceleration: a two-sample Mendelian randomization study

**DOI:** 10.1038/s41598-023-46308-4

**Published:** 2023-11-01

**Authors:** Yedong Huang, Xiaoyun Chen, Jingwen Ye, Huan Yi, Xiangqin Zheng

**Affiliations:** 1 College of Clinical Medicine for Obstetrics and Gynecology & Pediatrics, Fujian Medical University, Fuzhou, China; 2 National Key Gynecology Clinical Specialty Construction Institution of China, Fujian Provincial Key Gynecology Clinical Specialty, Fujian Maternity and Child Health Hospital, Affiliated Hospital of Fujian Medical University, Fuzhou, China; 3 Department of Respiratory Medicine, The First Affiliated Hospital of Fujian Medical University, Fuzhou, China; 4 Shanghai Sixth People’s Hospital Affiliated to Shanghai Jiao Tong University School of Medicine, Shanghai, China

**Keywords:** Genetics, Microbiology

## Abstract

The causal relationship between gut microbiota and DNA methylation phenotypic age acceleration remains unclear. This study aims to examine the causal effect of gut microbiota on the acceleration of DNA methylation phenotypic age using Mendelian randomization. A total of 212 gut microbiota were included in this study, and their 16S rRNA sequencing data were obtained from the Genome-wide Association Study (GWAS) database. The GWAS data corresponding to DNA methylation phenotypic age acceleration were selected as the outcome variable. Two-sample Mendelian randomization (TSMR) was conducted using R software. During the analysis process, careful consideration was given to address potential biases arising from linkage disequilibrium and weak instrumental variables. The results from inverse-variance weighting (IVW) analysis revealed significant associations (*P* < 0.05) between single nucleotide polymorphisms (SNPs) corresponding to 16 gut microbiota species and DNA methylation phenotypic age acceleration. Out of the total, 12 gut microbiota species exhibited consistent and robust causal effects. Among them, 7 displayed a significant positive correlation with the outcome while 5 species showed a significant negative correlation with the outcome. This study utilized Mendelian randomization to unravel the intricate causal effects of various gut microbiota species on DNA methylation phenotypic age acceleration.

## Introduction

The human gastrointestinal tract harbors an immensely large microbial community, encompassing an estimated range of 1000–1150 bacterial species collectively known as the gut microbiota (GM)^1^. In recent years, gut microbiota has emerged as a prominent focus of medical research and has been substantiated to be intricately associated with immune function, metabolism, and the development of various diseases^2, 3^. The composition of GM undergoes dynamic changes from infancy to adulthood and throughout the aging process in human individuals^4^. Therefore, gaining a comprehensive understanding of the profound association between gut microbiota and individual aging holds significant importance in the realms of anti-aging interventions and the prevention of age-related diseases.

The epigenetic clock, developed by Levin et al.^5^ in 2018, is a tool that utilizes gene methylation patterns to infer an individual's biological age. Existing research has demonstrated that biological age, as measured by the epigenetic clock, outperforms chronological age in assessing an individual's true aging status and predicting their lifespan^6^. DNA methylation phenotypic age acceleration, also known as epigenetic clock acceleration, serves as a biomarker reflecting an individual's aging status. It is commonly used to refer to the difference between an individual's biological age and chronological age^7^. Currently, it has been firmly established that GM is significantly associated with organismal aging and several age-related diseases^8–10^. However, the causal effects of GM on DNA methylation phenotypic age acceleration remain unclear.

Mendelian randomization (MR) is an epidemiological research method that utilizes genetic variation as instrumental variables (IVs) to investigate the causal associations between exposures and outcomes^11^. MR has gained widespread application in medical research in recent years due to its ability to mitigate confounding factors and reverse causality, which are often encountered in traditional epidemiological studies. Moreover, MR offers advantages such as cost-effectiveness, efficiency, and increased control over variables compared to randomized controlled trials (RCTs)^12, 13^. This study aims to investigate the causal impact of gut microbiota on DNA methylation phenotypic age acceleration using the analysis method of Mendelian randomization.

## Materials and methods

### Data sources and software preparation

The 212 gut microbiota datasets utilized in this study were obtained from GWAS database (http://gwas.mrcieu.ac.uk/datasets/). These data were derived from the sequencing information of the 16S rRNA of the gut microbiota in a total of 18,340 samples across 24 cohorts, as conducted by Kurilshikov et al^14^. The data on DNA methylation phenotypic age acceleration originates from a GWAS dataset based on epigenetic aging, specifically encoded as ebi-a-GCST90014292^15^. The data analysis was performed using the R software (version 4.1.3) and the TwoSampleMR package.

### Selection of instrumental variables for GM

The SNP selection for the gut microbiota followed the criterion of *P* < 1*10^–5^. To ensure adherence to Mendel's Second Law of Independent Assortment, which represents the principle of free combination, in this study, the criteria for linkage disequilibrium were set as follows: r^2^ < 0.001 and kb > 10,000. The SNPs filtered based on the aforementioned criteria will be used as IVs for subsequent analysis.

### Exclusion of weak instruments

To ensure the accuracy of the study results and the validity of the instrumental variable assumptions for Mendelian randomization, weak IVs were identified and removed based on the calculated F-statistic. The F-statistic is a statistical measure that reflects the strength of the association between IVs and the exposure factor. It is calculated using the formula: F = (β/SE) ^2^. SNPs are regarded as weak IVs and are excluded from the analysis if F < 10.

### Data analysis

Mendelian randomization analysis was conducted using the TwoSampleMR package in R software, employing the inverse-variance weighting (IVW) method. The regression results of IVW are used to determine whether there is a significant causal effect and directionality between the exposure and the outcome. To mitigate potential biases introduced by individual statistical methods, the weighted median (WM) and MR-Egger were employed as complementary analyses to the IVW. If the directions of the regression coefficients in the three aforementioned analyses are inconsistent, it indicates an unstable causal effect. In this step of the analysis, if the *P*-value of the IVW < 0.05, it is considered as a significant association between the exposure and the outcome.

### Heterogeneity and horizontal pleiotropy testing

In this study, Cochran's Q test and MR-Egger intercept test were employed to assess the heterogeneity and horizontal pleiotropy of the results, respectively. Heterogeneity represents the variability of causal effect estimates among each SNP. If heterogeneity is significant, it suggests an unstable causal effect between the exposure and the outcome. On the other hand, horizontal pleiotropy refers to the possibility of SNPs influencing the outcome through factors other than the exposure. If horizontal pleiotropy is significant, it indicates a violation of the exclusivity assumption in MR analysis. In both of the aforementioned tests, *P*-value < 0.05 is considered statistically significant.

### Ethics approval and consent to participate

This analysis of publicly available data does not require ethical approval.

## Results

### Data and detailed information

The GM data obtained from the GWAS database is used as the exposure variable, while DNA methylation phenotypic age acceleration is considered the outcome variable in this study. The GM data consists of a total of 212 sub-datasets, which cannot be fully presented in Table 1. For detailed information regarding the GM data, please refer to Supplementary Material 1 (Suppl. 1).

**Table 1:** Detailed information of the dataset used in this study.

Exposures/Outcomes	ID	Sample size	Author	Population	Year
Gut microbiota abundance	Not applicated	18,340	Kurilshikov	European and mixed	2021
DNA methylation PhenoAge Accel	ebi-a-GCST90014292	34,463	McCartney DL	European	2021

### IVs selection, linkage disequilibrium, and weak IVs exclusion

The following criteria were used to screen for SNPs: *P* < 1*10^–5^; linkage disequilibrium parameters: r^2^ < 0.001 and kb > 10,000. IVs with an F-statistic < 10 were excluded from the analysis. The F-statistics of each SNP and more detailed information can be found in Supplementary Material 2 (Suppl. 2), with F-statistics ranging from 14.90981 to 35.41665 for all SNPs.

### Results of MR

**Figure1:**
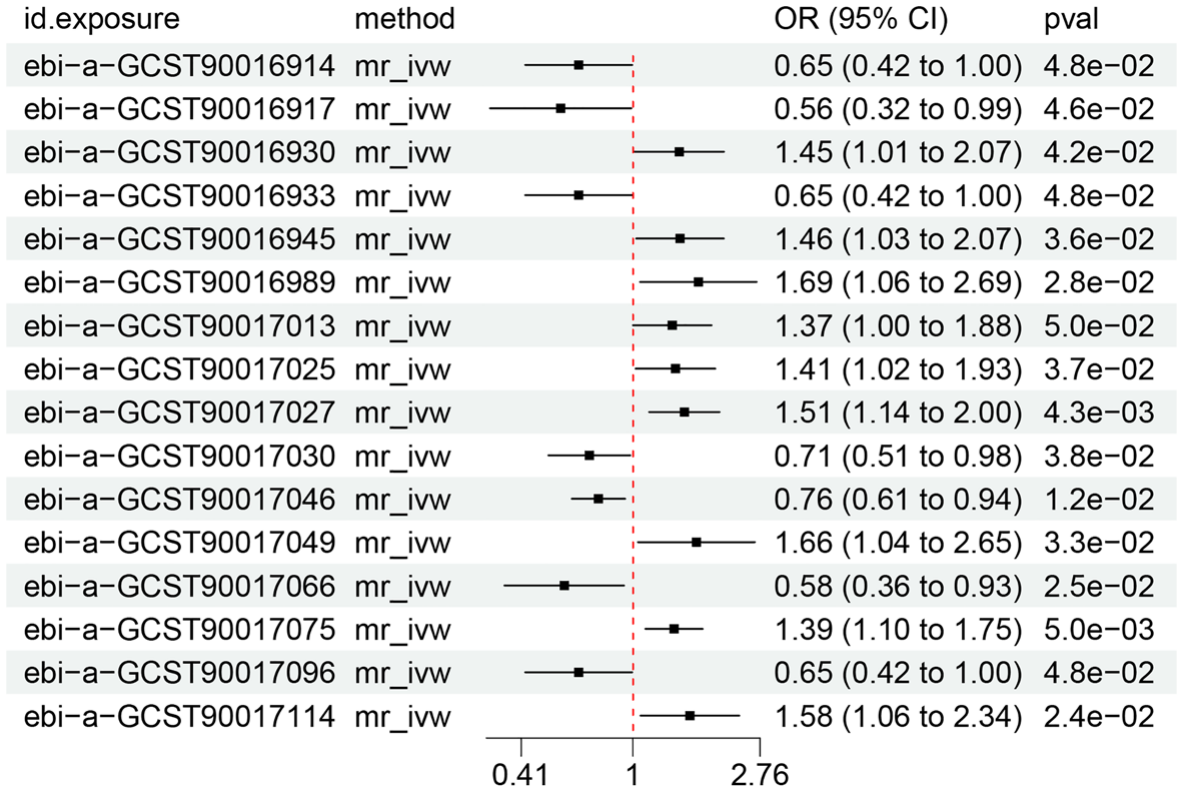
Forest plot for IVW results of 16gut microbiota.

As shown in Fig. 1, the analysis results of IVW indicate a potential causal association (*P* < 0.05) between the abundance of 16 GM species and DNA methylation phenotypic age acceleration. If the regression coefficients' directions from the Weighted median, MR-Egger and IVW methods are not consistent, it is considered an indication of an unstable causal effect and should be excluded from the final results. The detailed results of IVW and MR-Egger for the 16 GM species, including P-values and beta values are recorded in Table 2. Supplementary Material 3 (Suppl. 3) provides more detailed information about the 16 GM species.

**Table 2:** Detailed analysis results of IVW and MR-Egger of 16 gut microbiota.

ID.exposure	Exposure information	nsnp	Beta of IVW	Beta of MR-Egger	P value of IVW	P value of MR-Egger
ebi-a-GCST90016914	class Coriobacteriia id.809	14	− 0.432500	− 0.570237	0.047535	0.529630
ebi-a-GCST90016917	class Gammaproteobacteria id.3303	6	− 0.576390	0.347753	0.045634	0.728321
ebi-a-GCST90016930	family Christensenellaceae id.1866	27	0.370366	0.811673	0.042454	0.222573
ebi-a-GCST90016933	family Coriobacteriaceae id.811	14	− 0.432500	− 0.570237	0.047535	0.529630
ebi-a-GCST90016945	family Peptococcaceae id.2024	9	0.376363	1.000420	0.035539	0.055978
ebi-a-GCST90016989	genus Dorea id.1997	10	0.522970	0.820856	0.027930	0.249041
ebi-a-GCST90017013	genus Haemophilus id.3698	9	0.314864	− 0.010395	0.049975	0.978671
ebi-a-GCST90017025	genus Lachnospiraceae UCG001 id.11321	13	0.340038	0.841954	0.036977	0.273631
ebi-a-GCST90017027	genus Lachnospiraceae UCG008 id.11328	11	0.411210	1.285281	0.004303	0.122338
ebi-a-GCST90017030	genus Lactobacillus id.1837	10	− 0.347330	− 1.301232	0.037937	0.025866
ebi-a-GCST90017046	genus Rikenellaceae RC9 gut group id.11191	11	− 0.274830	0.050404	0.011965	0.944437
ebi-a-GCST90017049	genus Ruminiclostridium5 id.11355	11	0.507447	− 0.173350	0.033470	0.864088
ebi-a-GCST90017066	genus Ruminococcus torques group id.14377	9	− 0.547570	− 0.578948	0.024936	0.417178
ebi-a-GCST90017075	genus Tyzzerella3 id.11335	13	0.328764	0.536296	0.005028	0.417083
ebi-a-GCST90017096	order Coriobacteriales id.810	14	− 0.432500	− 0.570237	0.047535	0.529630
ebi-a-GCST90017114	phylum Firmicutes id.1672	16	0.455203	0.662726	0.024021	0.170209

### Heterogeneity and horizontal pleiotropy tests

**Figure 2:**
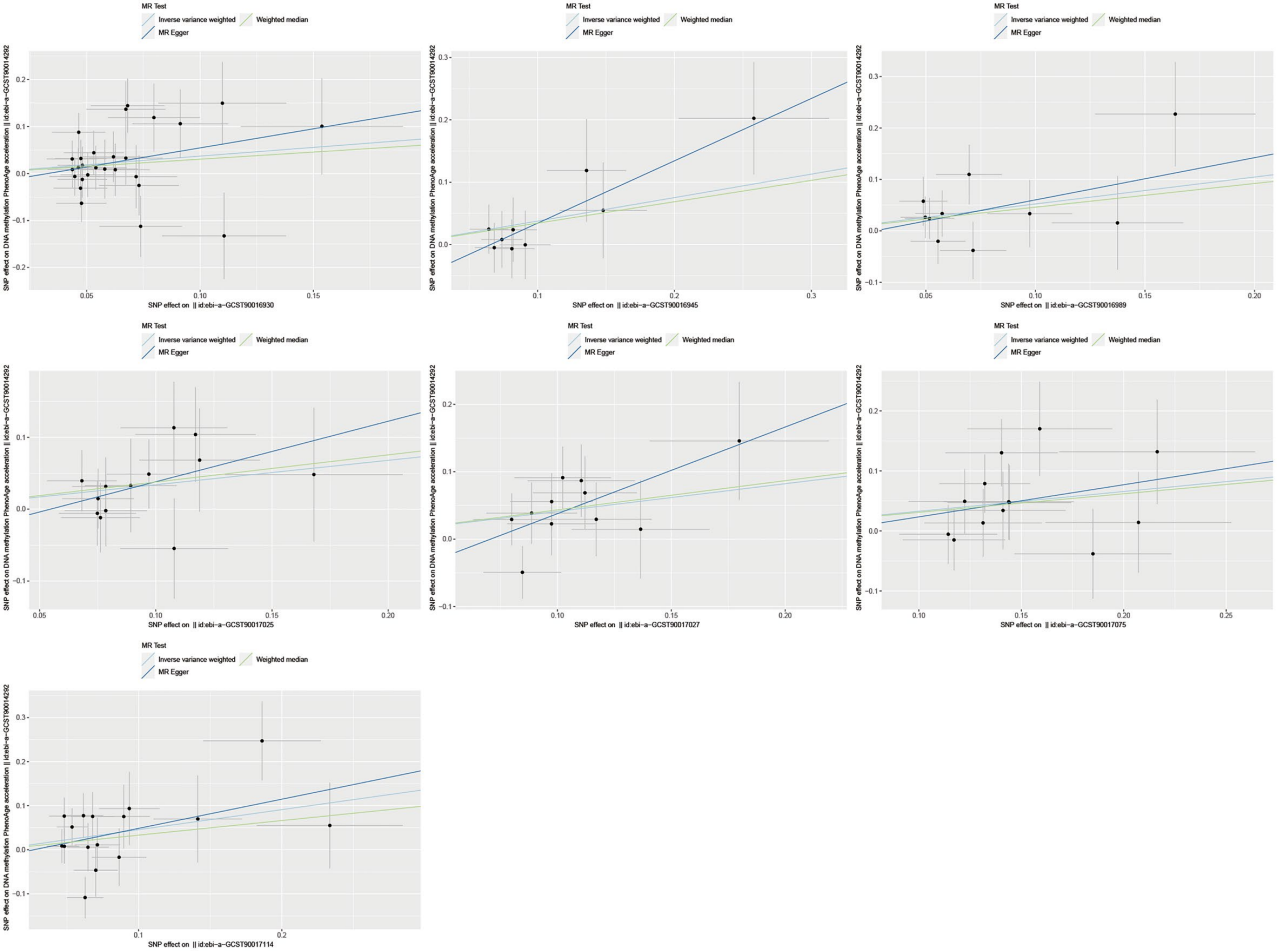
Scatter plots of 7 gut microbiota with forward direction.

**Figure 3:**
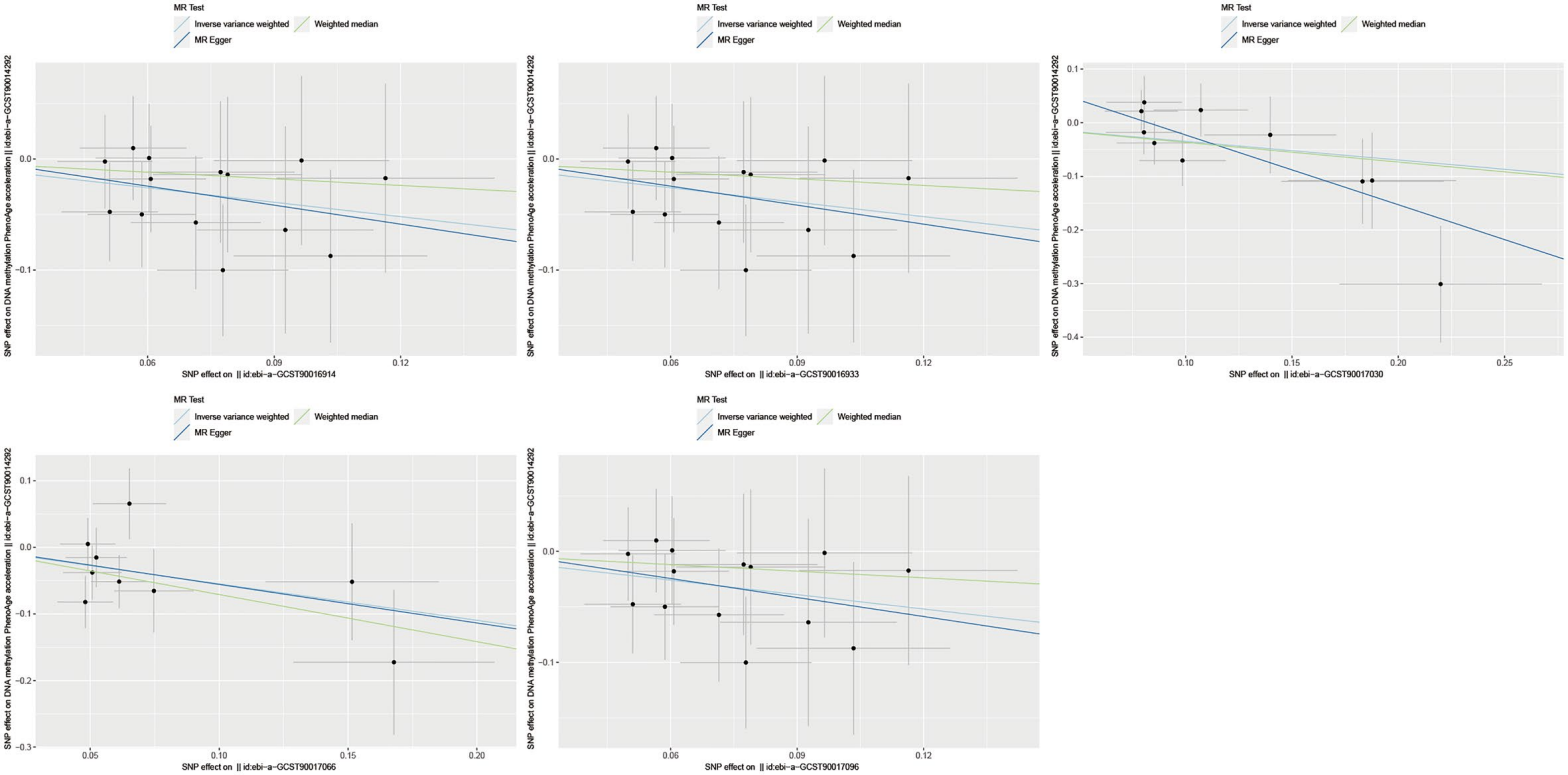
Scatter plots of 5 gut microbiota with negative direction. Footnote for Fig. 2 and Fig. 3: The positive or negative slope of the straight lines represents the direction of the causal effect, and three different colors are used to represent three different methods: light blue for IVW (Inverse Variance Weighted), dark blue for MR-Egger, and green for Weighted Median.

**Figure 4:**
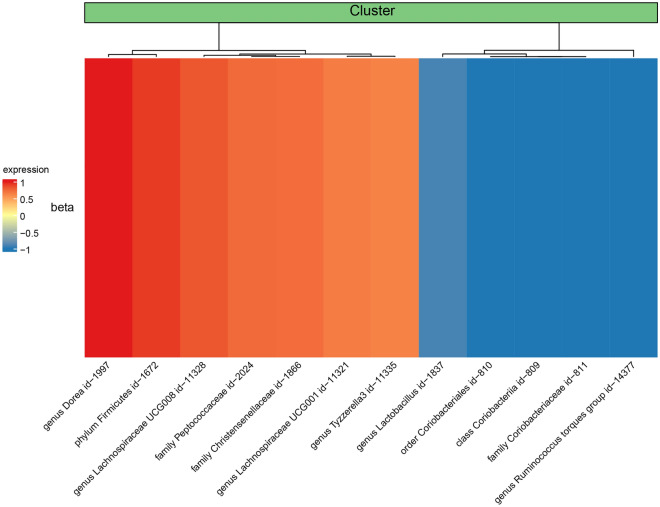
Heat map shows the beta ranking of 12 gut microbiota. Footnote: In the heatmap, the colors and shades represent the causal direction and effect size of the gut microbiota on the acceleration of the epigenetic clock, respectively. The dendrogram in the figure represents an unsupervised clustering analysis.

Heterogeneity and horizontal pleiotropy tests were conducted using Cochran's Q test and MR-Egger intercept test, respectively. If there is significant heterogeneity and horizontal pleiotropy (*P* < 0.05) observed for the SNPs corresponding to the GM in relation to the outcome, it indicates that the causal effect is not established. A total of 12 GM species showed relatively stable causal effects and passed the heterogeneity and horizontal pleiotropy tests (Table 3). As shown in Figs. 2 and 3, among the aforementioned 12 GM species, 7 species exhibited a significant positive correlation with the outcome (Fig. 2), while 5 species showed a significant negative correlation with the outcome (Fig. 3). Figure 4 presents a heatmap, generated using the complexheatmap package in R (version 4.1.3), displaying the sorted β-values of the 12 GM species in relation to DNA methylation phenotypic age acceleration.

**Table 3:** Detailed information of 12 gut microbiota with causal effect on the outcome.

ID	Microbiota	Beta	Direction
ebi-a-GCST90016914	class Coriobacteriia id.809	− 0.432500	Negative
ebi-a-GCST90016930	family Christensenellaceae id.1866	0.370366	Forward
ebi-a-GCST90016933	family Coriobacteriaceae id.811	− 0.432500	Negative
ebi-a-GCST90016945	family Peptococcaceae id.2024	0.376363	Forward
ebi-a-GCST90016989	genus Dorea id.1997	0.522970	Forward
ebi-a-GCST90017025	genus Lachnospiraceae UCG001 id.11321	0.340038	Forward
ebi-a-GCST90017027	genus Lachnospiraceae UCG008 id.11328	0.411210	Forward
ebi-a-GCST90017030	genus Lactobacillus id.1837	− 0.347330	Negative
ebi-a-GCST90017066	genus Ruminococcus torques group id.14377	− 0.547570	Negative
ebi-a-GCST90017075	genus Tyzzerella3 id.11335	0.328764	Forward
ebi-a-GCST90017096	order Coriobacteriales id.810	− 0.432500	Negative
ebi-a-GCST90017114	phylum Firmicutes id.1672	0.455203	Forward

Footnote: The β values here specifically denote the β of the IVW method.

## Discussion

Numerous studies have demonstrated a strong correlation between changes in GM and the aging process. However, there is a lack of definitive conclusions regarding the causal relationship between the two in the majority of these studies^16^. In the GM of healthy adults, the abundance of *Bacteroidetes* and *Firmicutes* phyla is generally higher, while *Actinobacteria* and *Proteobacteria* phyla are comparatively less abundant^8^,^17^. During the aging process, there is a gradual increase in the abundance of *Bacteroidetes* and *Proteobacteria* phyla, while the diversity of the gut microbiota tends to decrease^18^. Due to the complexity and diversity of the GM itself, identifying specific GM alterations associated with aging becomes challenging. Hence, this study aimed to explore the causal relationship between the GM and DNA methylation phenotypic age acceleration using MR analysis approach from the perspective of epigenetic clocks.

In this study, we identified seven specific GM species that exhibited significant positive causal effects on DNA methylation phenotypic age acceleration. These species were *Christensenellaceae*, *Peptococcaceae*, *Dorea*, *Lachnospiraceae UCG001*, *Lachnospiraceae UCG008*, *Tyzzerella3* and *Firmicutes*. A total of five GM species exhibited significant negative causal effects on DNA methylation phenotypic age acceleration. These species were *Coriobacteriia*, *Coriobacteriaceae*, *Lactobacillus*, *Ruminococcus torques group* and *Coriobacteriales*. Among them, *Coriobacteriia*, *Coriobacteriaceae*, and *Coriobacteriales* belong to the phylum *Actinobacteria*, while *Lactobacillus* and *Ruminococcus torques group* belong to the phylum *Firmicutes*. Therefore, based on the results of this study, it can be concluded that the causal effects of GM on DNA methylation phenotypic age acceleration are complex. In 2022, Kumar et al.^19^ utilized D-galactose to establish an animal model for accelerated aging and intervened by administering *Lactobacillus* through dietary supplementation. The findings revealed that *Lactobacillus* exhibited antioxidant potential in ameliorating the accelerated aging model. These results are consistent with our research, demonstrating a negative causal effect between the abundance of *Lactobacillus* and DNA methylation phenotypic age acceleration. In 2023, Liu et al.^20^ investigated the relationship between GM and longevity using Mendelian randomization. The results demonstrated a positive correlation between the abundance of *Coriobacteriaceae* and increased odds of longevity, which is consistent with the findings of our study.

In recent years, researchers have put forth the idea of fecal microbiota transplantation (FMT) in the context of longevity. FMT involves the transfer of gut microbiota from healthy and long-lived individuals to patients, aiming to potentially delay aging and promote longevity^21^^,^^22^. Although some studies have suggested a potential association between GM and the aging process^23–26^, the consistency of results among different studies is relatively poor. Therefore, further research is needed to comprehensively understand the impact of different GM species on the epigenetic clock. The findings of this study suggest that the causal effects of GM on the aging process are intricate, with different microbial taxa exerting distinct influences on DNA methylation phenotypic age acceleration. In conclusion, the findings of this study provide valuable insights for the clinical application of FMT and personalized treatments.

This study has several limitations. Firstly, the majority of the GWAS data used in this study were based on European individuals, which may introduce geographical and ethnic biases; Secondly, this study did not investigate the specific mechanisms through which different GM species influence the epigenetic clock. Further exploration is required using larger sample size and laboratory data to address these limitations.

## Conclusion

This study utilized Mendelian randomization to uncover the complex causal effects of different gut microbiota species on DNA methylation phenotypic age acceleration.

### Supplementary Information

Supplementary Information 1.

Supplementary Information 2.

Supplementary Information 3.

## Data Availability

The data that support the findings of this study are available from the corresponding author upon reasonable request.

## References

[1] Qin J (2010). A human gut microbial gene catalogue established by metagenomic sequencing. Nature.

[2] Lang JM (2018). Impact of individual traits, saturated fat, and protein source on the gut microbiome. Mbio.

[3] Kim D, Zeng MY, Nunez G (2017). The interplay between host immune cells and gut microbiota in chronic inflammatory diseases. Exp. Mol. Med..

[4] Badal VD (2020). The gut microbiome, aging, and longevity: A systematic review. Nutrients..

[5] Levine ME (2018). An epigenetic biomarker of aging for lifespan and healthspan. Aging (Albany NY)..

[6] Ashiqur RS (2021). Deep learning for biological age estimation. Brief. Bioinform..

[7] Ma Q (2022). Association between phenotypic age and mortality in patients with multivessel coronary artery disease. Dis. Markers..

[8] Biagi E (2017). The gut microbiota of centenarians: Signatures of longevity in the gut microbiota profile. Mech. Ageing Dev..

[9] Mangiola F, Nicoletti A, Gasbarrini A, Ponziani FR (2018). Gut microbiota and aging. Eur. Rev. Med. Pharmacol. Sci..

[10] O'Toole PW, Jeffery IB (2015). Gut microbiota and aging. Science.

[11] Lawlor DA, Harbord RM, Sterne JA, Timpson N, Davey SG (2008). Mendelian randomization: Using genes as instruments for making causal inferences in epidemiology. Stat. Med..

[12] Bowden J, Holmes MV (2019). Meta-analysis and Mendelian randomization: A review. Res. Synth. Methods..

[13] Birney E (2022). Mendelian Randomization. Cold Spring Harb. Perspect. Med..

[14] Kurilshikov A (2021). Large-scale association analyses identify host factors influencing human gut microbiome composition. Nat. Genet..

[15] McCartney DL (2021). Genome-wide association studies identify 137 genetic loci for DNA methylation biomarkers of aging. Genome Biol..

[16] Ling Z, Liu X, Cheng Y, Yan X, Wu S (2022). Gut microbiota and aging. Crit. Rev. Food Sci. Nutr..

[17] Mariat D (2009). The firmicutes/bacteroidetes ratio of the human microbiota changes with age. Bmc Microbiol..

[18] Claesson MJ (2011). Composition, variability, and temporal stability of the intestinal microbiota of the elderly. Proc. Natl. Acad. Sci. U.S.A..

[19] Kumar H (2022). Antioxidative potential of *Lactobacillus* sp. in ameliorating d-galactose-induced aging. Appl. Microbiol. Biotechnol..

[20] Liu X (2023). Mendelian randomization analyses reveal causal relationships between the human microbiome and longevity. Sci. Rep..

[21] Wang JW (2019). Fecal microbiota transplantation: Review and update. J. Formos. Med. Assoc..

[22] Barcena C (2019). Healthspan and lifespan extension by fecal microbiota transplantation into progeroid mice. Nat. Med..

[23] Claesson MJ (2012). Gut microbiota composition correlates with diet and health in the elderly. Nature.

[24] Biagi E (2010). Through ageing, and beyond: gut microbiota and inflammatory status in seniors and centenarians. PLoS ONE..

[25] Ticinesi A (2017). Aging gut microbiota at the cross-road between nutrition, physical frailty, and sarcopenia: Is there a gut-muscle axis?. Nutrients.

[26] Jackson MA (2016). Signatures of early frailty in the gut microbiota. Genome Med..

